# Ex Vivo Evaluation of Cementless Acetabular Cup Stability Using Impact Analyses with a Hammer Instrumented with Strain Sensors

**DOI:** 10.3390/s18010062

**Published:** 2017-12-27

**Authors:** Antoine Tijou, Giuseppe Rosi, Philippe Hernigou, Charles-Henri Flouzat-Lachaniette, Guillaume Haïat

**Affiliations:** 1 Laboratoire de Modélisation et de Simulation Multi-Echelle, CNRS, UMR CNRS 8208, 61 Avenue du Général de Gaulle, 94010 Créteil, France; antoine.tijou@u-pec.fr; 2 Laboratoire de Modélisation et de Simulation Multi-Echelle, UMR CNRS 8208, Université Paris-Est, 61 Avenue du Général de Gaulle, 94010 Créteil, France; Giuseppe.rosi@u-pec.fr; 3 Service de Chirurgie Orthopédique et Traumatologique, Hôpital Henri Mondor AP-HP, CHU Paris 12, Université Paris-Est, 51 Avenue du Maréchal de Lattre de Tassigny, 94010 Créteil, France; philippe.hernigou@aphp.fr (P.H.); charles-henri.flouzat-lachaniette@aphp.fr (C.-H.F.-L.); 4 Équipe 10, Groupe 5, IMRB U955, INSERM/UPEC, 51 Avenue du Maréchal de Lattre de Tassigny, 94010 Créteil, France

**Keywords:** total hip arthroplasty, implant stability, acetabular cup, impact

## Abstract

The acetabular cup (AC) implant stability is determinant for the success of cementless hip arthroplasty. A method based on the analysis of the impact force applied during the press-fit insertion of the AC implant using a hammer instrumented with a force sensor was developed to assess the AC implant stability. The aim of the present study was to investigate the performance of a method using a hammer equipped with strain sensors to retrieve the AC implant stability. Different AC implants were inserted in five bovine samples with different stability conditions leading to 57 configurations. The AC implant was impacted 16 times by the two hammers consecutively. For each impact; an indicator *I_S_* (respectively *I_F_*) determined by analyzing the time variation of the signal corresponding to the averaged strain (respectively force) obtained with the stress (respectively strain) hammer was calculated. The pull-out force *F* was measured for each configuration. *F* was significantly correlated with *I_S_* (R^2^ = 0.79) and *I_F_* (R^2^ = 0.80). The present method has the advantage of not modifying the shape of the hammer that can be sterilized easily. This study opens new paths towards the development of a decision support system to assess the AC implant stability.

## 1. Introduction

Total hip replacement (THR) has become a technique widely used in the clinic. Cementless implants, which are inserted within bone tissue using the press-fit procedure [1,2,3], are more and more used in hip arthroplasty [4,5,6]. However, there remain risks of failure, which may have dramatic consequences. Aseptic loosening is one of the main causes of failure for THR [7,8,9] and depends in particular on the quality of the implant primary stability, which determines the osseointegration process [10]. The long-term surgical success depends, among other factors, on the initial implant fixation for which a compromise should be found between: (i) excessive stresses at the bone-implant interface, that may lead to bone necrosis [11], and (ii) high relative micromotions at the bone-implant interface, that may lead to the presence of fibrous bone tissue around the implant surface, thus hampering osseointegration processes [10,12]. For these reasons, a compromise regarding the implant primary stability should be found in order to optimize the long term AC implant surgical success. 

The implant properties such as geometry and surface treatment, as well as the surgical protocol (reaming and impaction) influence the AC implant primary stability. Currently, surgeons listen the noise induced by the impact of the hammer on the ancillary [13] or rely on tactile information to evaluate the AC implant stability and to adapt their strategy related to the impact procedure (including the number and the energy of the impacts). With the aim to reach a suitable stability and to decrease the risk of per-operative bone fractures [14], a compromise should be found regarding the force and the number of impacts. However, quantitatively assessing implant stability remains difficult in the operative room.

X-ray [15] and magnetic resonance imaging [16] based technique have limitations to retrieve implant stability because of the artefacts caused by the presence of metal, which does not allow to accurately determine bone properties around the implant surface. Therefore, biomechanical approaches have been developed. Vibrational analyses are used in dental implantology to assess dental implant stability [17]. Different techniques have been developed to evaluate the stability of the femoral stem such as vibrational approaches [18,19,20] or a torsional rigidity measurements [21]. However, much less studies have been carried out to investigate the stability of the acetabular cup (AC) implant. Vibrational analyses were used to detect the AC loosening [22,23,24] and the implant stability [25]. Recently, a technique based on the analysis of the sound produced by the impact between the hammer and the ancillary was developed in order to control the AC fixation [26]. However, it remains difficult to quantitatively assess the AC primary stability in the operative room.

An alternative method consisting in measuring the time dependence of the force applied between the hammer and the ancillary during impacts has been developed by our group in order to derive information related to the AC implant stability. The contact duration was first found to be a useful indicator to follow the AC implant insertion [27] using reproducible mass drops. Another signal processing method using the estimation of the impact momentum [28] was found to allow the estimation of the AC primary stability [29]. With the aim to be used eventually intraoperatively in a patient specific manner, a hammer was then instrumented with a piezoelectric force sensor screwed on the impact surface of the hammer. The technique was adapted to predict the AC implant stability in vitro [30] and then in a cadaveric study [31]. Numerical studies were performed to understand the phenomena occurring during the impact [32,33]. However, the presence of a protuberance on a face of the hammer (corresponding to the piezoelectric sensor), as well as sterilization related issues currently prevents using such approach in the operating room. 

The purpose of the present study is to examine the performances of an impact hammer equipped with three strain gauges glued on the hammer surface in order to assess AC implant stability in vitro. The results will be compared with results obtained using the hammer instrumented with a force sensor similar as the one employed in [30]. 

## 2. Materials and Methods

### 2.1. Bone Specimen and Acetabular Cup Implant

**Figure 1:**
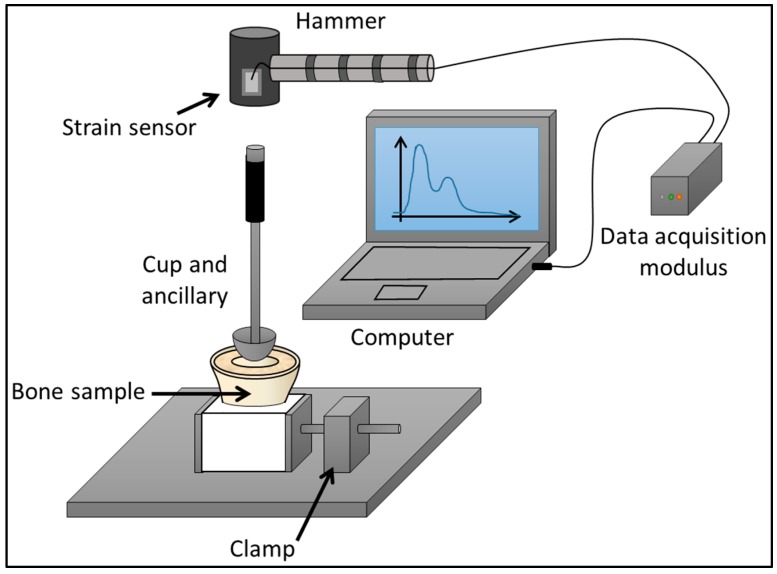
Schematic representation of the set-up employed for the impaction procedure of the acetabular cup implant into the bone sample.

Five bovine femurs have been retrieved from a butcher shop and prepared similarly as what has been done in [29,30,31]. Briefly, for each bone sample, the proximal epiphysis was cut and embedded in a fast hardening resin (polymer SmoothCast 300, Smooth-On, Easton, PA, USA). As shown in Figure 1 and similarly as what was done in [28], the bone sample was maintained by a clamp in order to position the upper surface of the trabecular bone region horizontally, which facilitates the AC implant insertion. Titanium alloy (TiAl6V4) AC implants (Ceraver, Roissy, France) with four diameters (49, 51, 53 and 55 mm) were used. Similarly as in clinical conditions, a dedicated ancillary was screwed in each AC implant and was handled by an experienced surgeon.

### 2.2. Experimental Set-Up for Impaction

**Figure 2:**
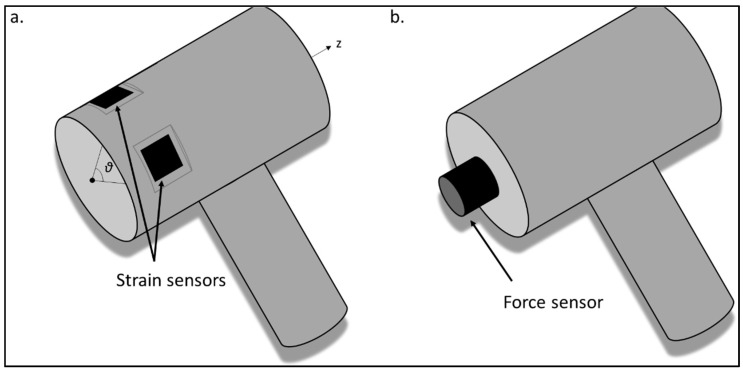
Schematic representation of (**a**) the strain hammer’s head instrumented with the three strain sensors and (**b**) the force hammer’s head instrumented with the force sensor.

Two similar hammers (ZEPF Medical Instruments, Seitingen-Oberflacht, Germany) shown in Figure 2, with a mass of 680 g and a head’s diameter equal to 40 mm, were used for the AC implant impactions. The first hammer is called in what follows the “strain hammer”. Three dynamic piezoelectric strain sensors (PI Ceramic, Lederhose, Germany) having a piezoelectric coefficient *K* equal of 500 pm/V were glued around its head close to the impact surface, as shown in Figure 2a, which defines the coordinate angle *θ* and the axis *Z*. The surface of the hammer’s head was slightly machined in order to obtain a planar surface allowing to secure the attachment between the strain sensors and the hammer body. All sensors were located in planes containing the axis *Z* of the hammer’s head. Moreover, the normal of the plane containing the three sensors corresponds to lines contained in planes defined by *θ* = 0°, 90° and 180° respectively. The three sensors were connected in series in order to average the three radiofrequency (rf) signals corresponding to the three sensors. 

The second hammer (see Figure 2b) (denoted “force hammer” in what follows) is instrumented with a dynamic piezoelectric force sensor (208C05, PCB Piezotronics, Depew, NY, USA) screwed in the center of one of the impact face, similarly as what has been done in [29,30,31].

The time variation of the strain and force signals obtained with the strain hammer (corresponding to the averaged signal obtained with the three strain gauges) and with the force sensor during each impact was recorded using a data acquisition module (NI 9234, National Instrument, Austin, TX, USA) with a sampling frequency of 51.2 kHz and a resolution of 24 bits.

### 2.3. Signal Processing

Two dedicated signal processing techniques were necessary in order to extract quantitative information from the rf signals obtained with the force and strain hammers respectively. 

The same signal processing technique as the one employed in [29,30,31] was used herein in order to determine a quantitative indicator *I_F_* from the rf signal obtained with the force hammer. For each impact the indicator *I_F_* was determined using the expression:

(1) $$I_{F}=\frac{1}{A_{0}*\left(t_{2}-t_{1}\right)}\int_{t_{1}}^{t_{2}}A\left(t\right).dt$$

where *A*(*t*) corresponds to the time variation of the force recorded by the force sensor, *t*_1_ = 0.27 ms and *t*_2_ = 0.82 ms, similarly as what has been done in [30]. *A*_0_ was chosen equal to 1100 N in order to obtain values of *I_F_* comprised between 0 and 1, for normalization purposes.

The signal processing technique employed to extract a quantitative indicator *I_s_* from rf signals obtained with the strain hammer was slightly different. First, the three rf signals obtained from the three strain gauges were averaged, leading to an averaged signal denoted *s*(*t*). Second, a third-order low-pass Chebyshev filter with a cut-off frequency (CF) equal to 5.12 kHz was applied to *s*(*t*) in order to remove high-frequency components, leading to a signal noted *S*(*t*). Third, the indicator *I_s_* was determined for each impact using:

(2) $$I_{s\;}=\frac{1}{S_{0}*\left(t_{4}-t_{3}\right)}\int_{t_{3}}^{t_{4}}S\left(t\right).dt$$

where *t*_3_ = 0.27 ms and *t*_4_ = 0.52 ms. *S*_0_ was chosen equal to 0.19 in order to obtain values of *I_s_* comprised between 0 and 1. The values chosen for *t*_3_, *t*_4_ and CF will be discussed in the discussion section. The data were analyzed with Matlab (The Mathworks, Natick, MA, USA).

### 2.4. Pull-Out Mechanical Test

Similarly as in [29,30,31], a pull-out tangential mechanical test was carried out in order to evaluate the AC stability. A force was applied at the top end of the ancillary perpendicularly to its axis whereas the bone sample was firmly attached. A numerical dynamometer (DFX2-050-NIST, AMETEK, Elancourt, France) was used in order to measure the maximum value *F* of the force necessary to extract the AC from the bone.

### 2.5. Experimental Protocol

**Figure 3:**
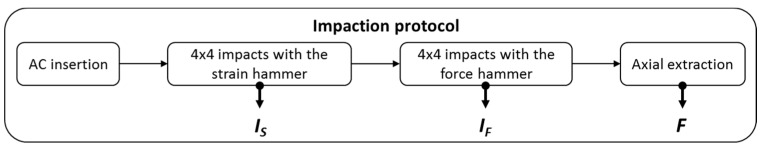
Schematic description of the experimental protocol realized for each configuration corresponding to a given acetabular cup diameter (ACD) and a given bone cavity diameter (BCD).

A hemispherical cavity with a diameter equal to 47 mm was initially drilled in trabecular bone. Then, an impaction procedure was carried out with a 48 mm diameter AC implant and consisted in four steps as described in Figure 3. Firstly, the AC implant was inserted within the bone cavity until achieving a suitable stability without damaged the host bone. Secondly, the ancillary was impacted four successive times using the strain hammer, with the constraint that the maximum of the sensor’s elongation should be comprised between −1 and −3 nm, which corresponds to a weak impact energy compared to the ones employed to insert AC implant. The value of the indicator *I_s_* was determined using Equation (2) for each of the four impacts and then averaged in order to obtain the average value $\tilde{I_{s}}$ of the indicator *I_s_* for the corresponding configuration. In order to assess the reproducibility of the estimation of $\tilde{I_{s}}$, the measurement was carried out three additional times and the average and the standard deviation values (denoted $I_{S}^{M}$ and $I_{S}^{SD}$) obtained for the four values of $\tilde{I_{s}}$ were determined.

The same experiment was then performed with the force hammer. Similarly as what was done in [29,30,31], the maximum of the force should be comprised between 2500 N and 4500 N, which corresponds to an impact with a weak amplitude. For each of the four successive impacts, the value of the indicator *I_F_* was determined using Equation (1) and the average value $\tilde{I_{F}}$ was calculated. In order to assess the reproducibility of the estimation of $\tilde{I_{F}}$, the measurement was carried out three additional times and the average and the standard deviation values (denoted $I_{F}^{M}$ and $I_{F}^{SD}$) obtained for the four values of $\tilde{I_{F}}$ were determined. The choices of the number of impacts (four) and the number of repetitions of the procedure (four) will be discussed in the discussion section. 

Finally, a tangential pull-out test was performed following the protocol defined in Section 2.4 with the aim to assess the AC stability. The impaction procedure described above was repeated with the same AC implant diameter (ACD) and the same bone cavity diameter (BCD) as long as the operator felt that an acceptable implant stability could be obtained again without damaging the surrounding bone tissue. Then, an AC implant with an ACD equal to 50 mm was employed with the same cavity. The values of ACD and BCD were chosen so that the interference fit was equal to 1 or 3 mm [6,9,30]. The BCD was then increased up to a value of 49 mm and the impaction procedure described above was again carried out with an ACD equal to 50 mm. The impaction procedure was repeated with the same values of ACD and BCD as long as the operator felt that a good implant stability could be obtained without damaging bone tissue. Again, an implant with an ACD equal to 53 mm was then used based on the same considerations as the one described above. The BCD was then increased successively to values equal to 51 mm and then to 53 mm following the same aforementioned protocol. All bone samples that underwent fracture were excluded from the study.

### 2.6. Statistical Analyses

Relationships between $I_{S}^{M}$ and *F* (resp. $I_{F}^{M}$ and *F*) were studied using linear regression analyses. The same analyses were employed to investigate the relationship between $I_{S}^{M}$ and $I_{F}^{M}$. 

## 3. Results

57 impaction procedures were performed, leading to 57 values of $I_{S}^{M}$, $I_{S}^{SD}$, $I_{F}^{M}$, $I_{F}^{SD}$ and *F*. Table 1 shows the number of impaction procedures realized for the five bone samples and each combination of BCD and ACD. 

The total number of impaction procedures considered varies according to the sample, because (i) of the different bone quality and (ii) the different cavities were not realized in the exact same configuration (since the cavities were realized manually, similarly as in the operating room). 

**Figure 4:**
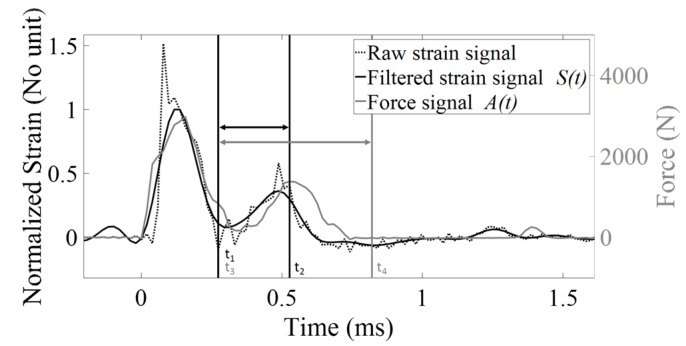
Time-variation of the signal obtained with the force hammer (grey solid line) and of the signal obtained with the strain hammer (black dotted line) for the same configuration corresponding to sample #1, BCD = 51 mm, ACD = 54 mm. The filtered rf signal derived from the strain hammer is also shown with a solid black line. For this configuration *F* = 95.4 N, *I_s_* = 0.52 and *I_F_* = 0.73.

Figure 4 shows the time dependence of the force measured with the impact hammer (grey line) and of the strain measured the strain hammer (black dotted line) for the same configuration corresponding to sample #1, BCD = 51 mm, ACD = 54 mm. The filtered rf signal derived from the strain hammer is also shown with a solid black line. The qualitative variation of the signal obtained with the force hammer and of the filtered signal obtained with the strain hammer is similar. Both signals exhibit a maximum amplitude around 0.15 ms and then a secondary maximum around 0.50 ms. 

**Figure 5:**
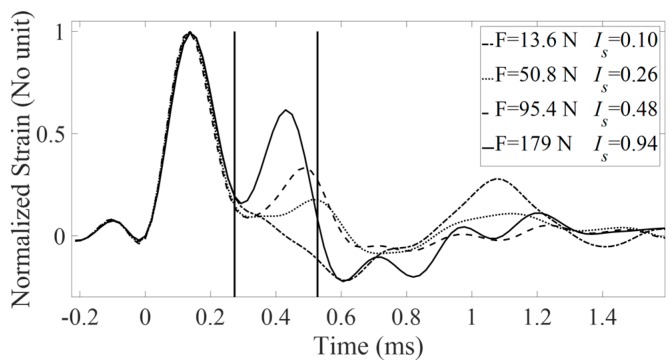
Normalized and filtered rf signals obtained with the strain hammer for various configurations corresponding to four different values of the AC implant stability. The solid line corresponds to sample #2, BCD = 53 mm and ACD = 54 mm. The dashed line corresponds to sample #1, BCD = 51 mm and ACD = 54 mm. The dotted line corresponds to sample #5, BCD = 47 mm and ACD = 48 mm. The dashed dotted line corresponds to sample #4, BCD = 47 mm and ACD = 50 mm. The values of the AC implant stability *F* and of the indicators *I_s_* are indicated for each configuration.

Figure 5 shows different normalized and filtered rf signals obtained with the strain hammer for various configurations corresponding to four different values of the AC implant stability. Figure 5 shows that the amplitude of the rf signal in the time window used for the determination of *I_s_* increases when the AC implant stability *F* increases. 

**Figure 6:**
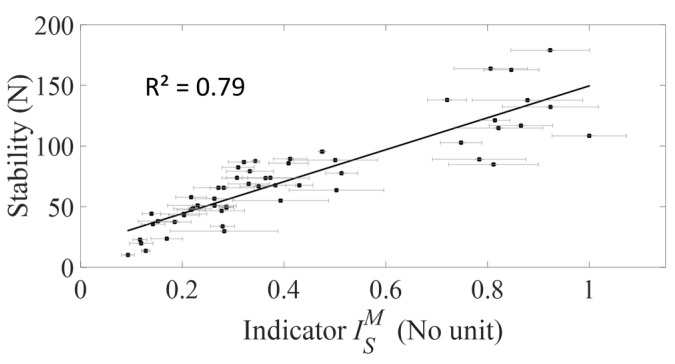
Variation of the averaged value and the standard deviation of the indicator $I_{S}^{M}$ obtained with the strain hammer and the tangential stability *F* for all data pooled corresponding to the five bone samples and to all impaction procedures.

Figure 6 shows the variation of the AC implant stability *F* as a function of the average value of the indicator $I_{S}^{M}$ obtained with the strain hammer for all data pooled. The standard deviation $I_{S}^{SD}$ of indicators $\tilde{I_{s}}$ values are represented by the error bars. A linear regression between the averaged value of the indicator $I_{S}^{M}$ and the tangential stability *F* shows a significant correlation (R^2^ = 0.79, *p*-value < 0.001). 

**Figure 7:**
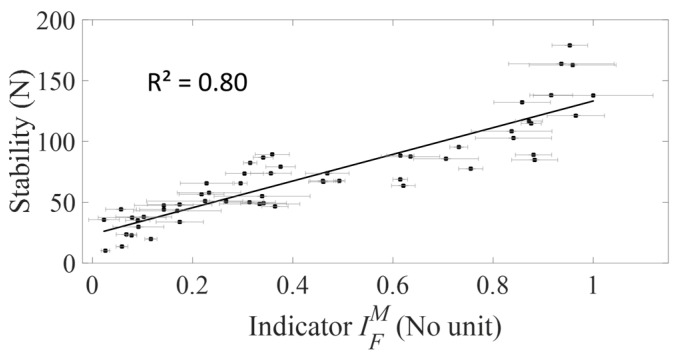
Variation of the averaged value and the standard deviation of the indicator $I_{F}^{M}$ obtained with the force hammer and the tangential stability *F* for all data pooled corresponding to the five bone samples and to all impaction procedures.

Figure 7 shows the variation of the AC implant stability *F* as a function of the average value of the indicator $I_{F}^{M}$ obtained with the force hammer for all data pooled. The standard deviation $I_{F}^{SD}$ of indicators $\tilde{I_{F}}$ values are represented by the error bars. A linear regression between the averaged value of the indicator $I_{F}^{M}$ and the tangential stability *F* shows a significant correlation (R^2^ = 0.80, *p*-value < 0.001).

**Figure 8:**
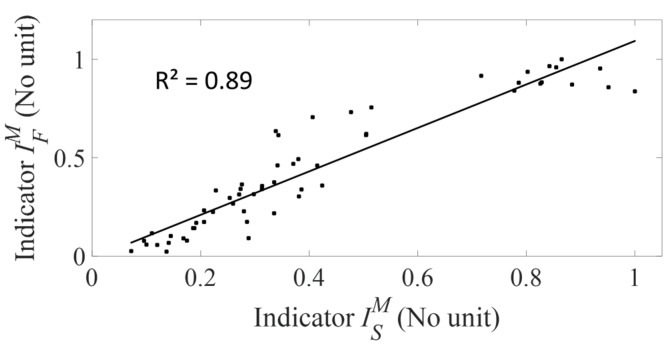
Variation of the averaged value of the indicator $I_{S}^{M}$ obtained with the strain hammer and of the averaged value of the indicator $I_{F}^{M}$ obtained with the force hammer for all data pooled corresponding to the five bone samples and to all impaction procedures.

Figure 8 shows the relationship between the averaged values of $I_{S}^{M}$ and $I_{F}^{M}$ for all data pooled. A linear regression shows a significant correlation between $I_{S}^{M}$ and $I_{F}^{M}$ (R^2^ = 0.89, *p*-value < 0.001).

## 4. Discussion

The originality of the present study is to demonstrate the feasibility of using a hammer equipped with strain sensors in order to retrieve information on the stability of an AC implant in vitro. To do so, a comparison between the results obtained with a hammer instrumented with a force sensor similar as the one employed in [30] (called force hammer) and with a hammer equipped with three strain sensors is investigated. The significant correlation between the indicator $I_{S}^{M}$ and the tangential stability *F* (R^2^ = 0.79, see Figure 6) constitutes a validation of this approach. Moreover, Figure 8 shows that the indicator $I_{S}^{M}$ and the indicator $I_{F}^{M}$ are significantly correlated (R^2^ = 0.88), which indicates that the information retrieved using both methods is qualitatively similar, as suggested in Figure 4. The averaged value of the standard deviation of the indicator $I_{S}^{SD}$ ($I_{F}^{SD}$, respectively) is equal to 0.41 (respectively 0.44), which corresponds to a precision expressed in percentage equal to 11% (respectively 10%). The precision error is therefore comparable when using both instrumented hammers. 

The results obtained in Figure 7 corresponding to the force hammer are in good agreement with the results obtained [30,31] in comparable situations. The determination coefficient corresponding to the linear regression between the indicator $I_{F}^{M}$ and the tangential stability *F* obtained herein is equal to 0.80 while it was equal to 0.83 in our previous in vitro study [30] and to 0.69 in a cadaveric study [31]. 

The results shown in Figure 5 can be explained by considering the variation of the resonance frequency of the bone-implant when the implant stability varies. When the contact area between bone and the implant increases, it induces an increase of the stiffness of the bone-implant system and thus of the resonance frequency, which explain the variation of the signal measured by the instrumented hammer. This mechanism had already been described experimentally [30,31], numerically [33] and analytically [28] by our group.

**Figure 9:**
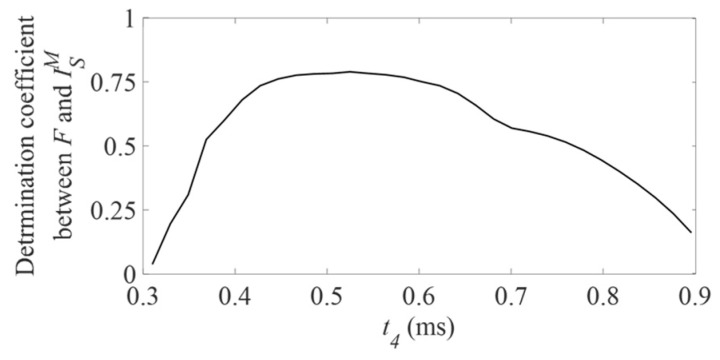
Variation of the determination coefficient R^2^ between *F* and $I_{S}^{M}$ as a function of the value of *t*_4_.

The impact momentum corresponding to both hammers was defined using approximately the same interval as in the previous in vitro and cadaveric studies [28,29,30,31]. The value of *t*_3_ = 0.27 ms was chosen equal to value of *t*_1_, because of the similarity of the rf signals obtained with both hammers investigated in the present study. The value of *t*_2_ = 0.82 ms was chosen equal to the value chosen in [30] as mentioned in Section 2.3. Changing the values of *t*_2_ between 0.72 ms and 0.92 ms did not affect significantly the results (less than 5% difference for R^2^ between *F* and $I_{F}^{M}$). An optimization study was run to find the value of *t*_4_ that maximizes the correlation coefficient between $I_{S}^{M}$ and *F*. For each value of *t*_4_ comprised between 0.30 ms and 0.90 ms, the values of the indicator $I_{S}^{M}$ were computed for all configurations and the determination coefficient R^2^ between *F* and $I_{S}^{M}$ was calculated. The results are shown in Figure 9. The maximum value of R^2^ was found for *t*_4_ = 0.52 ms. Changing the values of *t*_4_ between 0.42 ms and 0.62 ms did not affect significantly the results (less than 5% difference for R^2^ between *F* and $I_{S}^{M}$). 

**Figure 10:**
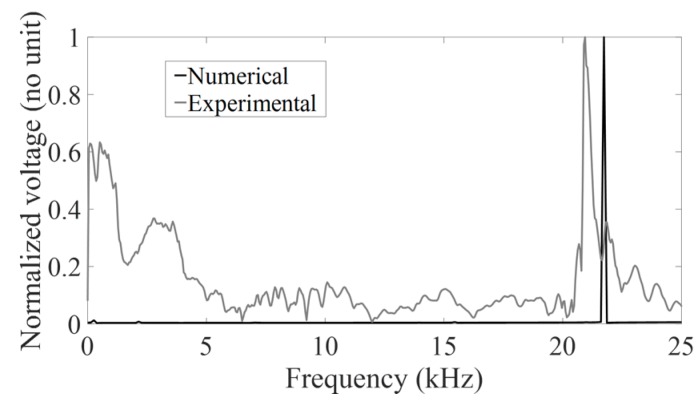
Frequency response measured at the electrode of the finite element model of the hammer (black line) and the spectrum of a representative signal *s*(*t*) (grey line).

As shown in Figure 2, the strain sensors were attached to the lateral surface of the strain hammer. Therefore, the strain sensors are likely to be sensitive not only to the impacts, but also to the natural resonance frequency of the hammer itself. The grey line in Figure 10 shows the frequency spectrum of a typical rf signal *s*(*t*) where a resonance frequency is obtained around 21 kHz. In order to understand the physical determinant of this resonance frequency, a simple 3-D finite element model was developed in the frequency domain taking into account the geometry of the strain hammer using the COMSOL software (Comsol AB, Stockholm, Sweden). The multiphysical numerical code solves the elasto-dynamic equations for the hammer, while the sensors are modelled as piezoelectric materials. At these frequencies, the wavelength of electromagnetic waves is much larger than the thickness of the piezoelectric elements. Thus, a quasistatic assumption is used for the electric fields. The model has 49,272 tetrahedral quadratic elements, and it is solved for 222,241 degrees of freedom in the frequency domain. The black line in Figure 10 shows the frequency response of the hammer in terms of voltage measured at the electrodes, the solicitation being a Gaussian surface at the impact surface. This simulation does not take into account the real impact since the aim is to identify the modes of vibration of the hammer, as excited by a harmonic source. The resonance frequency obtained around 22 kHz corresponds to the free variation of the hammer head (data not shown), which does not carry any useful information on the impact. The difference between the experimental and numerical resonance frequency may be explained by the uncertainty on the exact geometry and material properties. In order to filter this resonance frequency, a low-pass Chebyshev filter with a cut-off frequency equal to 5.1 kHz was chosen. We verified (data not shown) that changing the value of the CF frequency between 4 kHz and 7 kHz did not affect significantly the results (less than 2% difference for R^2^).

Another advantage of carrying out numerical simulations is that they can also be used to provide some guidelines in the choice of the number and of the positioning of the strain transducers. In particular, we avoided to locate strain sensors at the lower side of the hammer head, near to the handle in order to minimize the effect of the normal frequencies of the hammer, which allows to minimize the sensitivity with respect to the point of impact. However, further extensive studies should be carried out in the future to optimize the choice of the location of the sensors.

As described in Section 2.5, the measurement protocol consists in realizing four successive impacts, and to repeat this procedure four times, leading to a total number of 16 impacts. The choice of these impact numbers was made to reach the following compromises. First, the number of impacts, equal to four, was chosen to find a compromise between: (i) obtaining a sufficient number of measurement to correct possible errors due to impact conditions using averaging and (ii) limiting the total number of impacts to minimize the time necessary to carry out future measurements in the operating room. Second, according to the previous studies [30,31], the number of the measurement protocol repetitions performed in order to assess the stability was chosen equal to four. This number is high enough to evaluate the reproducibility of the measurement but low enough to avoid the occurring of possible fracture or alteration of the bone-implant relationship. The number of impaction procedures (see Table 1) realized for each bone sample and each value of bone cavity diameter (BCD) and acetabular cup diameter (ACD) was chosen empirically by the surgeon to obtain enough configurations without damaging bone tissue. 

**Figure 11:**
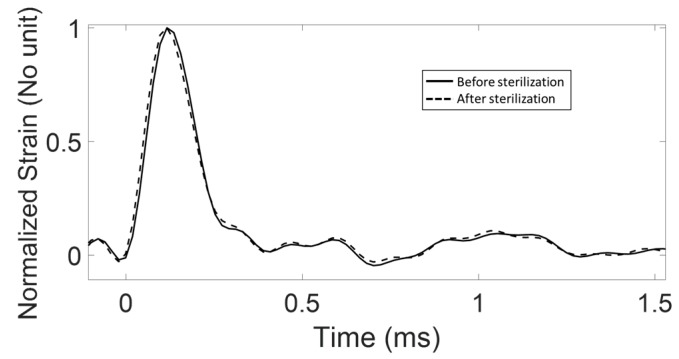
Representation of two signals obtained using the strain hammer in the same configuration before (solid line) and after the application of a sterilization procedure in an autoclave (dashed line).

The first difference between the two hammers lies in their geometry. The force hammer has a protuberance due to the force sensor, which makes it difficult to use it in the clinic because the surgeon has to impact the ancillary on this protuberance, which is not easy and may disrupt the surgeon’s gesture. Conversely, the geometry of the strain hammer is similar to existing orthopedic hammers and the impacting face is flat and large enough. The second difference between the two hammers lies in the fact that the force sensor cannot be sterilized because the presence of electronic components inside the sensor, according to the specifications provided by the manufacturer. Meanwhile, the strain hammer can be sterilized because the piezoelectric gauges can handle up to 175 °C (Curie temperature equal to 350 °C), according to the manufacturer. Figure 11 shows two signals obtained with the same configuration and with the same strain hammer before and after an autoclave cycle (one hour with a maximum temperature of 134 °C and a maximum pressure of 0.21 MPa) leading to the sterilization of the hammer, similarly as what is done in the clinic. The results show that the two signals are almost identical, the difference being due to the reproducibility of the measurements.

This study has several limitations. First, human periacetabular bone and bovine femoral bone have different biomechanical properties. We chose to work with real bone samples instead of bone mimicking phantoms following our previous work [30] because it allows to obtain a variability on bone properties that is likely to be encountered in the clinical situation. However, it would also be interesting to consider bone mimicking phantoms because it allows to work under standardized conditions, which should be done in future studies. A study was performed in cadavers in order to validate the force hammer [31] and a forthcoming study will be realized with cadavers with the aim to validate the strain hammer. Second, the bone cavity was not measured precisely for each impaction series. However, the aim of this study was not to relate the indicator to the cavity diameter. Third, the operator dependence is an important issue regarding clinical use of the hammer and must be investigated. Fourth, the influence of damping related to the presence of soft tissue was not investigated herein. Fifth, the sterilization study could be improved. Indeed, a future study including more sterilization cycles should be considered. Sixth, only five bovine bone samples were considered in this study. However, the total number of configurations was equal to 57, which is justified by the following power analysis. Namely, a power analysis of the variation of the determination coefficient R^2^ obtained between the variables $I_{S}^{M}$, $I_{F}^{M}$ on the one hand and *F* on the other hand has been performed using the software XLSTAT (Addinsoft, Bordeaux, France) in order to compute the power of the linear regression with a number of observations (N) equal to 57. The calculation uses the non-central Fischer distribution with the following parameters: the first degree of freedom is equal to 1 (which corresponds to the number of variables), the second degree of freedom is equal to 57 and the non-centrality parameter is equal to f^2^ × N = 8.55 (f^2^ is chosen equal to 0.15 and corresponds to a medium effect size). The significance level is chosen equal to 0.05. For these parameters, the computed power is equal to 0.82, which indicates that the number of configurations was sufficient to perform a statistically robust linear regression between our variables ($I_{S}^{M}$, $I_{F}^{M}$ and *F*).

## 5. Conclusions

This study constitutes a first validation of using a hammer equipped with piezoelectric strain sensors in order to retrieve information on the AC implant stability. This approach has the advantage of not modifying the geometry of usual orthopedic hammers currently used in operating room and to allow the hammer sterilization using an autoclave, thus opening the path for a potential use in the operating room. Other advantages of the approach lies in the fact that the hammer could be sterilized more easily and that it does not alter the surgical protocol. 

## Figures and Tables

**Table 1:** Number of impaction procedures realized for each bone sample and for the different value of bone cavity diameter (BCD) and acetabular cup diameter (ACD).

BCD (mm)	ACD (mm)	Sample #1	Sample #2	Sample #3	Sample #4	Sample #5	Total
47	48	1	2	2	1	3	9
47	50	0	0	0	1	1	2
49	50	2	3	2	2	3	12
49	52	0	0	0	1	0	1
51	52	3	3	2	2	3	13
51	54	3	3	0	1	1	8
53	54	3	3	2	2	2	12
Total		12	14	8	10	13	57
